# Decreases in Circulating Concentrations of Long-Chain Acylcarnitines and Free Fatty Acids During the Glucose Tolerance Test Represent Tissue-Specific Insulin Sensitivity

**DOI:** 10.3389/fendo.2019.00870

**Published:** 2019-12-17

**Authors:** Elina Makarova, Marina Makrecka-Kuka, Karlis Vilks, Kristine Volska, Eduards Sevostjanovs, Solveiga Grinberga, Olga Zarkova-Malkova, Maija Dambrova, Edgars Liepinsh

**Affiliations:** ^1^Pharmaceutical Pharmacology, Latvian Institute of Organic Synthesis, Riga, Latvia; ^2^Faculty of Pharmacy, Riga Stradins University, Riga, Latvia; ^3^Faculty of Biology, University of Latvia, Riga, Latvia

**Keywords:** glucose tolerance test, insulin resistance, long-chain acylcarnitines, free fatty acids, type 2 diabetes

## Abstract

**Background:** Insulin plays a pivotal role in the regulation of both carbohydrate and lipid intermediate turnover and metabolism. In the transition from a fasted to fed state, insulin action inhibits lipolysis in adipocytes, and acylcarnitine synthesis in the muscles and heart. The aim of this study was to measure free fatty acid (FFA) and acylcarnitine levels during the glucose tolerance test as indicators of tissue-specific insulin resistance.

**Results:** Insulin release in response to glucose administration decreased both FFA and long-chain acylcarnitine levels in plasma in healthy control animals by 30% (120 min). The glucose tolerance test and [^3^H]-deoxy-D-glucose uptake in tissues revealed that high fat diet-induced lipid overload in *C57bl/6N* mice evoked only adipose tissue insulin resistance, and plasma levels of FFAs did not decrease after glucose administration. In comparison, *db/db* mice developed type 2 diabetes with severely impaired insulin sensitivity and up to 70% lower glucose uptake in both adipose tissues and muscles (skeletal muscle and heart), and both plasma concentrations of FFAs and long-chain acylcarnitines did not decrease in response to glucose administration.

**Conclusions:** These results link impaired adipose tissue insulin sensitivity with continuous FFA release in the transition from a fasted to postprandial state, while a blunted decrease in long-chain acylcarnitine levels is associated with muscle and heart insulin resistance.

## Introduction

Currently, the main diagnostic markers of type 2 diabetes mellitus used in clinical practice are related to measurements of impaired glucose metabolism (1, 2), but these markers do not reveal disturbances in insulin sensitivity in different tissues. Recently, assessments of dyslipidemia or fasting triglycerides and total cholesterol have been included as components of a comprehensive medical evaluation for diabetes and therapeutic efficiency (1). Despite extensive studies, clinical validation of other suggested circulating lipid-related metabolites has been insufficient to accept these markers into clinical practice (3–9).

The profile of energy metabolism is significantly changing during the fed-and-fasted cycle, switching from the utilization of fatty acids (FAs) in a fasted state to carbohydrates in a fed state. The inability of metabolism to adapt to a particular nutritional state is referred to as metabolic inflexibility, which is characterized by abnormally high concentrations of metabolites for a particular nutritional state (10). The plasma levels of FFAs and acylcarnitines increase proportionally to the duration of fasting as a result of an increase in lipolysis, and synthesis of acylcarnitines is decreased by the activation of insulin signaling during the fed or postprandial state (11). In healthy subjects, fasted-state insulin levels are low, and evaluation of FAs and acylcarnitine concentrations in a fasted state does not properly characterize insulin sensitivity (12). Instead, in the postprandial or fed state, high concentrations of free fatty acids (FFAs) and acylcarnitines are considered as markers of insulin resistance, characterizing insulin inability to stop the breakdown of triglycerides in adipocytes as well as to inhibit CPT-1-dependent FA metabolism in muscles and the heart (3, 13).

Long-chain acylcarnitines are formed in mitochondria from free L-carnitine and acyl-CoA moieties (14, 15). Because long-chain FAs are the main energy substrates in the skeletal muscles and the heart, these tissues are considered essential contributors to the long-chain acylcarnitine pool in plasma (16, 17). Increased plasma levels of various acylcarnitines were found in experimental animal models of insulin resistance (16) and in patients with obesity, impaired glucose tolerance and type 2 diabetes (11, 18–22). However, previous studies did not evaluate the tissue-specific impairment of insulin action and reported inconclusive associations of plasma acylcarnitine levels with glucose disposal.

The postprandial state is not well-defined; therefore, for better control of glucose load, the oral glucose tolerance test (GTT) or a controlled meal is suggested for diagnostic purposes. The GTT is routinely applied as a diagnostic method, and additional measurements of FA intermediates in the fasted state and 2 h after the glucose administration are feasible in clinical conditions. Additionally, measurements during the GTT are not just steady-state measurements but also a characterization of the transition from a fasted to a postprandial state, which reflects the ability of organisms to adapt to glucose load and the capability to decrease FA flux and metabolism. Therefore, in this study, we tested whether the development of insulin resistance in muscle and adipose tissue is associated with an impaired decrease in lipid intermediates in the transition from a fasted to postprandial state. We used HFD-fed mice as a model of early stage insulin resistance and *db/db* mice that develop type 2 diabetes with severely impaired glucose metabolism (23, 24). We measured the concentrations of plasma FFAs and acylcarnitines after glucose administration in HFD-fed and *db/db* mice and evaluated whether these measurements, in addition to the GTT, could serve as biomarkers of tissue-specific insulin resistance.

## Materials and Methods

### Animals

Twenty male *C57bl/6N* mice (7 weeks old, Envigo, Netherlands), 10 male diabetic *db/db* (*BKS*.Cg-+Lepr/+Lepr/OlaHsd) mice and age-matched 10 control *db/L* mice (7 weeks old, Envigo) were housed under standard conditions (21–23°C, relative humidity 50 ± 10%, 12 h shifted light-dark cycle) with unlimited access to food and water. The experimental procedures were carried out in accordance with the guidelines of the European Community (2010/63/EU), local laws and policies and were approved by the Latvian Animal Protection Ethical Committee, Food and Veterinary Service, Riga, Latvia. Performed studies are reported in accordance with the ARRIVE guidelines (25, 26). Mice were adapted to local conditions for 2 weeks before the start of treatment and were housed in individually ventilated cages with softwood bedding material (five animals per cage). Based on statistical power analysis we used 10 animals per group to observe significant decrease in plasma acylcarnitine levels by at least 30%. C*57bl/6N* mice were randomly divided into two groups. The control group (*n* = 10, with average body weight of 20.9 ± 0.4 g) received a standard diet (R70 diet, Lantmännen, Sweden) for 8 weeks. To induce insulin resistance, the other group (*n* = 10, with average body weight of 20.6 ± 0.4 g) received a high fat diet (HFD) (21% fat and 0.15% cholesterol, Western RD, Special Diets Services, UK) for 8 weeks.

Blood glucose was measured using a MediSense Optium (Abbott Diabetes Care, UK) blood glucose meter and strips. Plasma insulin concentrations were determined with a rat/mouse insulin ELISA kit (Millipore, USA). To perform the GTT, the drug naïve mice were fasted overnight. Then, the glucose solution (0.5 g/kg of body weight) was administered intraperitoneally, and blood samples were then drawn from the tail vein at 0 (fasting), 1 and 2 h after glucose administration. The whole blood glycated hemoglobin A1C (HbA1C) concentration was measured with an automated hemoglobin testing system (DCA Vantage Analyzer, Siemens, USA). Plasma FFA concentrations were evaluated using a commercially available enzymatic assay kit from WAKO (Germany) according to the manufacturer's instructions.

### Measurement of Acylcarnitine Levels by UPLC/MS/MS

The concentrations of acylcarnitines in the plasma samples were determined with a UPLC MS/MS method using a Waters Acquity liquid chromatography system and Waters Quattro Micro or Waters Xevo TQ-S mass spectrometer, as previously described (17, 27).

### Measurement of Glucose Uptake

To determine the insulin-sensitive glucose uptake in tissues, 1 μCi of 2-[1,2-^3^H]-deoxy-D-glucose ([^3^H]-DOG, specific activity, 60 Ci/mmol) was administered subcutaneously (s.c.) to the mice at 1 h 50 min after glucose administration. After 10 min, the mice were sacrificed by decapitation, and the heart, skeletal muscle (1:5, w/v in MilliQ water) and adipose tissue homogenates (1:3, w/v in MilliQ water + 1% Igepal) were prepared. The content of [^3^H]-DOG in the samples was determined by liquid scintillation.

### Data Analysis

The results are expressed as the mean ± standard error mean (SEM). After assessment of the normality of data, statistically significant differences in the mean values were tested by unpaired *t*-test; paired *t*-test was used to compare the differences from baseline. The differences were considered significant when *p* < 0.05. The data were analyzed using GraphPad Prism statistical software (GraphPad Inc., USA).

## Results

**Figure 1:**
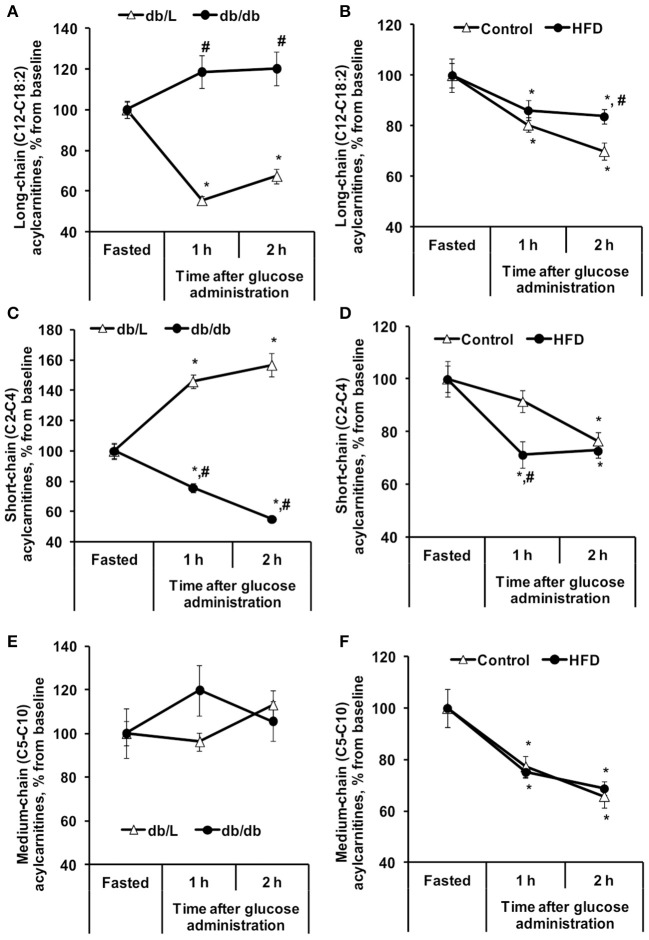
Changes in plasma acylcarnitine concentrations in *db/db* and HFD-fed mice during the GTT. Long-chain **(A,B)**, short-chain **(C,D)**, and medium-chain **(E,F)** acylcarnitine concentrations were determined in fasted blood plasma samples at 1 and 2 h after glucose administration in *db/L* and *db/db*, control and HFD-fed mice. Values are represented as the average ± SEM of 5–10 animals. *Significantly different from the fasted state (paired *t*-test, *p* < 0.05). ^#^Significantly different from the control group (unpaired *t*-test, *p* < 0.05).

**Figure 2:**
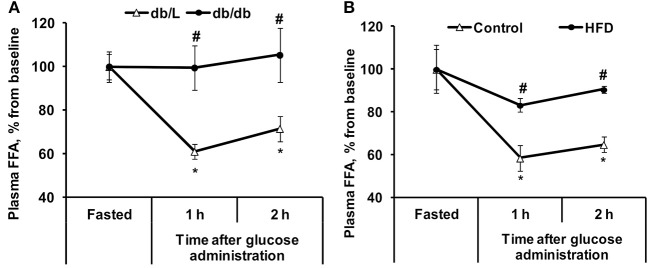
Changes in plasma FFA concentrations in *db/db* and HFD-fed mice during the GTT. FFA **(A,B)** concentrations were determined in fasted blood plasma samples at 1 and 2 h after glucose administration in *db/L* and *db/db*, control and HFD-fed mice. Values are represented as the average ± SEM of 5–10 animals. *Significantly different from the fasted state (paired *t*-test, *p* < 0.05). ^#^Significantly different from the control group (unpaired *t*-test, *p* < 0.05).

To measure the insulin-induced effect on lipid metabolism, the changes in plasma FFA and acylcarnitine levels after glucose administration in the GTT were monitored (Table 1, Figures 1, 2). Entire acylcarnitine profile is included in the Supplementary Table 1. Plasma levels of long-chain acylcarnitine in fasted HFD-fed and *db/db* mice compared with the corresponding controls were 1.3- and 2.5-fold lower, respectively. During the GTT at 2 h after glucose administration, the levels of long-chain acylcarnitines in plasma were significantly reduced by up to 33% in both the control *db/L and C57bl/6N* mice (Figures 1A,B). In the *db/db* mice, the plasma long-chain acylcarnitine concentration did not decrease during the GTT (Figure 1A). In comparison to chow-fed *C57bl/6N* mice, the plasma levels of long-chain acylcarnitine at 2 h after glucose administration in the HFD-fed mice decreased by 14–16%, and this level of long-chain acylcarnitine was significantly different from both the fasting level and the corresponding control group level at 2 h after glucose administration (Figure 1B).

**Table 1:** Plasma short-chain, medium-chain, and long-chain acylcarnitine concentrations in experimental mouse models of insulin resistance and type 2 diabetes in the fasted state.

	**Control**	**HFD**	***db/L***	***db/db***
**SHORT-CHAIN AC, nmol/ml**				
C2	53 ± 3.5	36 ± 1.8*	20 ± 0.9	28 ± 1.5*
C3	0.39 ± 0.03	0.29 ± 0.03*	0.18 ± 0.01	0.015 ± 0.01
C4	0.40 ± 0.02	0.53 ± 0.03*	0.20 ± 0.01	0.64 ± 0.07*
C5	0.18 ± 0.01	0.16 ± 0.01	0.18 ± 0.01	0.41 ± 0.05*
Sum	54 ± 3.6	37 ± 1.8*	20 ± 0.9	29 ± 1.5*
**MEDIUM-CHAIN AC, nmol/ml**				
C6	0.12 ± 0.01	0.11 ± 0.01	0.08 ± 0.01	0.05 ± 0.00^*^
C8	0.02 ± 0.00	0.02 ± 0.00	0.02 ± 0.00	0.01 ± 0.00^*^
C10	0.02 ± 0.00	0.03 ± 0.00	0.02 ± 0.00	0.01 ± 0.00^*^
C12	0.04 ± 0.00	0.05 ± 0.01	0.04 ± 0.00	0.02 ± 0.00^*^
Sum	0.34 ± 0.03	0.32 ± 0.02	0.30 ± 0.02	0.48 ± 0.05^*^
**LONG-CHAIN AC, nmol/ml**				
C14	0.13 ± 0.01	0.14 ± 0.01	0.14 ± 0.01	0.04 ± 0.00^*^
C16	0.65 ± 0.03	0.47 ± 0.06^*^	0.44 ± 0.02	0.13 ± 0.01^*^
C18:0	0.10 ± 0.00	0.07 ± 0.00^*^	0.05 ± 0.00	0.03 ± 0.00^*^
C18:1	0.93 ± 0.05	0.71 ± 0.03^*^	0.54 ± 0.03	0.27 ± 0.01^*^
C18:2	0.06 ± 0.00	0.02 ± 0.00^*^	0.05 ± 0.00	0.01 ± 0.00^*^
Sum	1.90 ± 0.09	1.46 ± 0.10^*^	1.26 ± 0.05	0.51 ± 0.02^*^
**TOTAL AC, nmol/ml**				
	56 ± 3.6	39 ± 1.9^*^	22 ± 0.9	29 ± 1.6^*^

Values are represented as the average ± SEM of 9–10 animals.

* *Significantly different from the fasted state (unpaired t-test, p < 0.05)*.

Short-chain acylcarnitines, mainly acetylcarnitines, constitute 90–95% of the total plasma acylcarnitine content on average (Table 1). In the fasted state, the concentration of short-chain acylcarnitines in the plasma of control *C57bl/6N* mice was 54 ± 3.6 nmol/ml, and in HFD-fed mice, the plasma levels of short-chain acylcarnitines were 1.5-fold lower. Compared to control *C57bl/6N* mice, fasted *db/L* mice had 2.7-fold lower concentrations of short-chain acylcarnitine in plasma, but in *db/db* mice, the concentrations of short-chain acylcarnitines in plasma were 1.45-fold higher than those in *db/L* mice (Table 1). The levels of short-chain acylcarnitines in plasma at 2 h after glucose load were significantly decreased by 23, 27, and 45% in *C57bl/6N* control, HFD-fed and *db/db* mice, respectively (Figures 1C,D). Only *db/L* mice showed an increase in short-chain acylcarnitine plasma concentrations by 57% (Figure 1C).

In the fasted state, plasma levels of medium-chain acylcarnitines did not differ between the control and HFD-fed *C57bl/6N* mice, while in *db/db* mice, the plasma concentration of medium-chain acylcarnitines was increased by 1.6-fold compared to that in *db/L* mice (Table 1). The concentrations of plasma medium-chain acylcarnitines in *db/db* mice were similar to those in the *db/L* mice and did not change during the GTT (Figure 1E). In contrast, in control and HFD-treated *C57bl/6N* mice, medium-chain acylcarnitine levels were significantly decreased by 31% (Figure 1F). Thus, short-chain and medium-chain acylcarnitine levels were equally reduced in control and HFD-fed animals, but in *db/db* mice, no consistent association was observed between the changes in plasma short-chain or medium-chain acylcarnitine concentrations and the degree of insulin resistance.

In the healthy control *db/L* animals at 2 h after glucose administration, the concentration of plasma FFA decreased by 28% on average. In contrast, in the *db/db* mice with pronounced insulin resistance in adipose tissue, the FFA levels did not change during the GTT (Figure 2A). In chow-fed *C57bl/6N* mice, the decrease in plasma FFA concentration at 2 h after glucose load was 35%, but similarly to the plasma levels in *db/db* mice, in insulin-resistant HFD-fed mice, the plasma FFA levels were not significantly decreased during this time period (Figure 2B).

**Figure 3:**
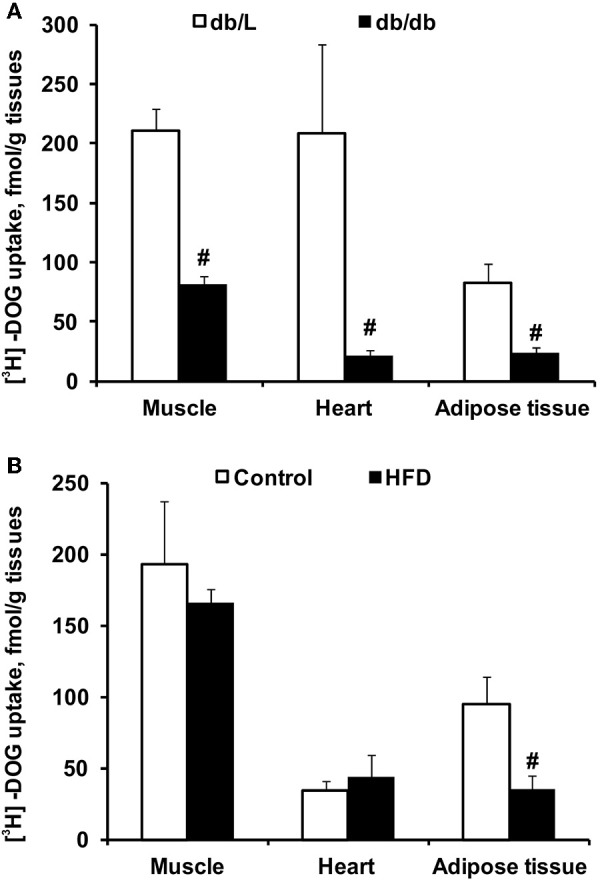
Tissue glucose uptake in *db/db* and HFD-fed mice. The uptake of ^3^H-DOG in the skeletal muscle, heart and adipose tissue in *db/L* and *db/db* **(A)**, control and HFD-fed **(B)** mice was measured 2 h after the GTT. Values are represented as the average ± SEM of 4–5 animals. ^#^Significantly different from the control group (unpaired *t*-test, *p* < 0.05).

The uptake of [^3^H]-DOG was assessed to evaluate the insulin sensitivity in various tissues. As shown in Figure 3A, the rate of glucose uptake in *db/db* mice was decreased in the heart, muscle and adipose tissue by 2- to 8-fold compared to db/L mice. In HFD-fed mice, glucose uptake was reduced by 2.7-fold only in adipose tissue, but it was not altered in the heart or muscle tissues (Figure 3B).

**Figure 4:**
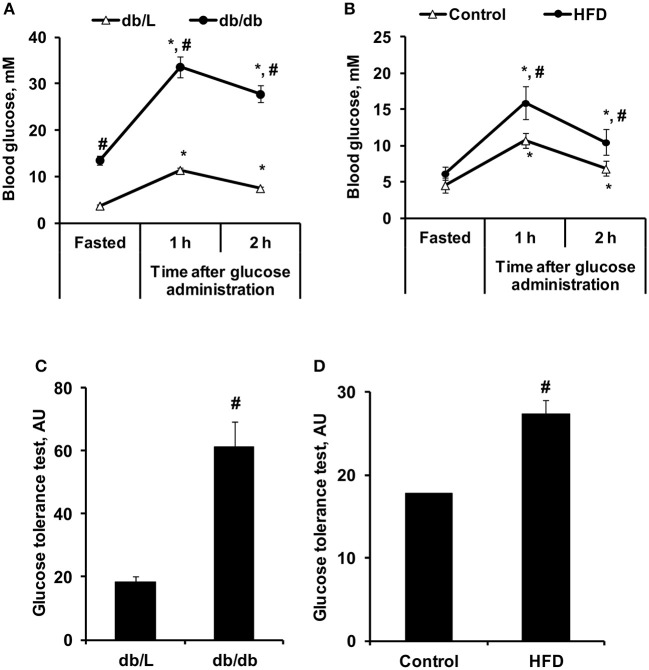
Changes in glucose concentrations during the GTT in control, HFD-fed, *db/L*, and *db/db* mice. Changes in glucose concentrations during the GTT **(A,B)** and the calculated AUC of the change from basal glucose levels **(C,D)** in *db/L* and *db/db*, control and HFD-fed mice. Values are represented as the average ± SEM of 10 animals. *Significantly different from the fasted state (paired *t*-test, *p* < 0.05). ^#^Significantly different from the control group (unpaired *t*-test, *p* < 0.05).

**Figure 5:**
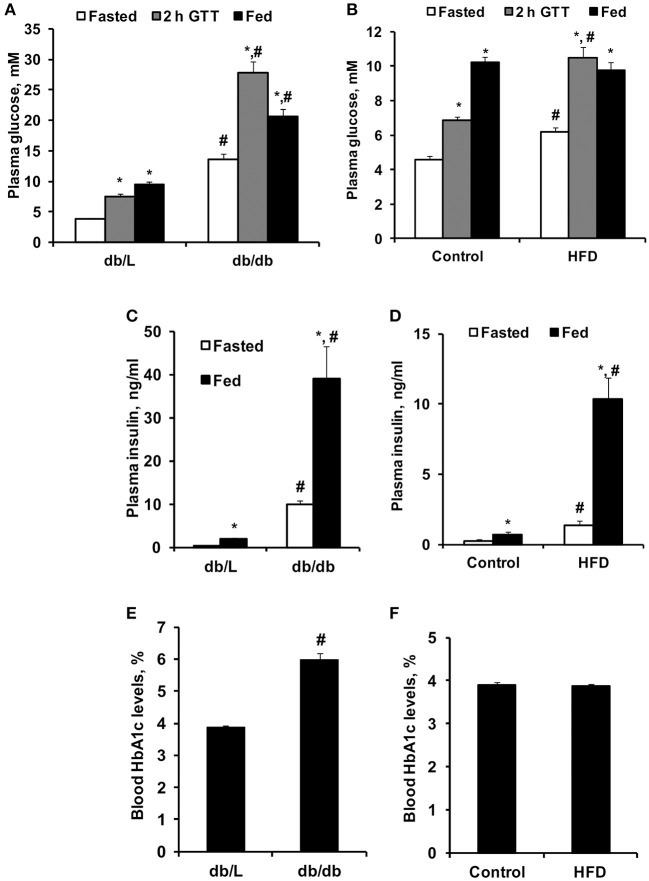
Concentrations of blood glucose, plasma insulin and HbA1c in control, HFD-fed, *db/L*, and *db/db* mice. Glucose concentrations in fasted and fed states and at 2 h after the glucose tolerance test (GTT) **(A,B)**; fed and fasted insulin plasma levels **(C,D)** and blood HbA1c levels **(E,F)** in *db/L* and *db/db*, control and HFD-fed mice. Values are represented as the average ± SEM of 5–10 animals. *Significantly different from fasted state (paired *t*-test, *p* < 0.05). ^#^Significantly different from the control group (unpaired *t*-test, *p* < 0.05).

To characterize the selected experimental models of insulin resistance and type 2 diabetes, conventional plasma metabolites were detected in the fed and fasted states, and the GTT was performed (Figures 4, 5). *Db/db* mice had considerably impaired glucose tolerance, and the area under the curve was 3-fold greater than that in *db/L* mice (Figures 4A,C). In comparison, after 8 weeks of HFD intake, the glucose tolerance in *C57bl/6N* mice was moderately impaired, and the area under the curve after the GTT was increased by 54% compared to the control group (Figures 4B,D). Overall, in HFD mice moderate but in *db/db* severe glucose intolerance and insulin resistance were observed.

As shown in Figure 5A, *db/db* mice had significantly increased fasted and fed state plasma glucose levels by approximately 2- and 4-fold, respectively, compared to db/L mice, as well as a 4-fold increase in plasma glucose concentration 1 and 2 h after the administration of glucose in the GTT. Administration of the HFD for 8 weeks induced only 35–54% higher glucose concentrations in the fasted state for up to 2 h after the administration of glucose in the GTT, while in the fed state, no significant differences in glucose concentrations were observed compared to control mice (Figure 5B). Pronounced hyperinsulinemia was observed in *db/db* and HFD-fed mice in the fasted and fed states compared to the respective control groups (Figures 5C,D). Thus, in *db/db* and HFD-fed mice, insulin concentrations were up to 20- and 14-fold higher than those in control mice, respectively (Figures 5C,D). HbA1c, as an indicator of long-term hyperglycemia in *db/db* mice, was increased by approximately 50% compared to control and *db/L* mice but was not elevated in HFD-treated mice (Figures 5E,F).

## Discussion

In the present study, we tested whether a decrease in circulating FFA and long-chain acylcarnitine concentrations after glucose administration in a GTT is associated with insulin sensitivity and can be used for the diagnosis of insulin resistance. In our study, insulin release in response to glucose administration similarly decreased FFA and long-chain acylcarnitine levels in plasma in healthy control animals by 30% (120 min). Here, we show that a blunted decrease in long-chain acylcarnitine plasma concentration during the GTT is associated with muscle-specific insulin resistance, while postprandial changes in plasma FFA levels reflect adipose tissue insulin sensitivity.

In healthy subjects, fasted-state insulin levels are low, and evaluation of acylcarnitine concentrations in a fasted state does not characterize insulin action (12) still, multiple clinical studies have been performed to assess circulating acylcarnitine levels in fasted subjects for the evaluation of insulin response. (11, 18–20). In the present study, fasted-state long-chain acylcarnitine levels were significantly decreased in both experimental models, with adipose and muscle tissue insulin resistance, compared to healthy controls. This decrease indicates on fasting hyperinsulinemia-induced inhibition of CPT1-mediated long-chain acylcarnitine synthesis (7, 27, 28). These observations are in line with a study by Schooneman et al., showing that the insulin sensitivity index did not correlate with fasted levels of plasma acylcarnitines, and improvement in insulin sensitivity in overweight individuals resulted in an increase in fasted state levels of individual acylcarnitine species (C2, C4OH, C10, C14:1, C16, C18:1) (29). Moreover, improved metabolic flexibility and insulin sensitivity in response to caloric restriction was related to higher fasting and postprandial differences in long-chain acylcarnitine and FFA concentrations (30). Overall, in the fasted state, when the plasma insulin level is comparably low, the measurements of long-chain acylcarnitine measurements can be used to characterize disorders of mitochondrial fatty acid oxidation (31), while this state is not appropriate to estimate the accumulation of long-chain acylcarnitines levels in relation to insulin resistance.

**Figure 6:**
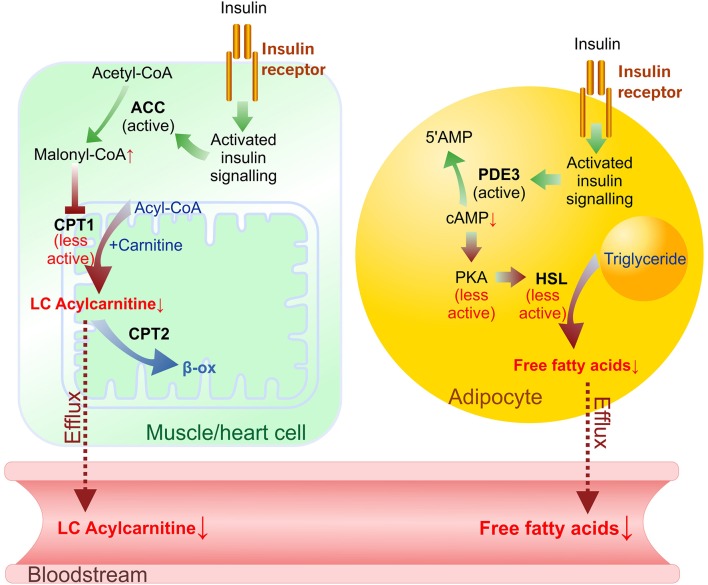
Insulin-induced effects on long-chain (LC) acylcarnitine synthesis in muscle/heart cells and lipolysis in adipocytes in the transition from fasted to postprandial (fed) state. Plasma levels of FFAs and LC acylcarnitines depend on acylcarnitine synthesis rate in muscles/heart and lipolysis rate in adipocytes. In muscle and heart cells activation of insulin signaling prevents ACC phosphorylation, stimulates malonyl-CoA synthesis and results in CPT-1 inhibition. In adipocytes cAMP-dependent suppression of lipolysis by insulin signaling involves activation of PDE3 (which degrades cAMP), releasing PKA from activation, and decreasing lipolysis through a reduction in the phosphorylation-mediated activation of HSL and perilipin. Green arrows—activation, red arrows—inhibited reactions. Dotted line—efflux. ACC, acetyl-CoA carboxylase; β-ox, β-oxidation; CPT, carnitine palmitoyltransferase; HSL, hormone-sensitive lipase; PKA, protein kinase A (cAMP-dependent protein kinase); PDE3, phosphodiesterase 3B.

In a postprandial state, the synthesis of acylcarnitines is inhibited by endogenous insulin (7, 28); therefore, the measurement of changes in acylcarnitine concentrations after controlled glucose load characterizes insulin sensitivity (Figure 6). Indeed, in our study, a significant, 30% decrease in long-chain acylcarnitine plasma levels during the GTT was detected in healthy control animals, while this decrease was less pronounced in HFD-treated mice with moderate insulin resistance, and no decrease was observed in the *db/db* mice with type 2 diabetes. In diabetic *db/db* mice, the observed hyperglycemia and markedly impaired glucose response during the GTT were related to the diminished rate of insulin-dependent glucose uptake in the heart, muscle and adipose tissues. In contrast, HFD-fed mice developed moderate changes in glucose homeostasis, and significantly impaired insulin sensitivity was observed in only adipose tissue. Considering that glycemia and glucose uptake were maintained in the presence of markedly elevated insulin levels, HFD mice had an early stage of tissue insulin insensitivity. Measurements of glucose uptake confirmed that *db/db* mice developed severely impaired insulin sensitivity and glucose uptake in skeletal muscle and heart tissue. Interestingly, a limited effect on the concentration of acylcarnitines was measured in HFD-treated mice even without significantly affecting glucose uptake. Our findings are in line with previous studies in which in insulin-resistant subjects, glucose-stimulated insulin release was not able to adequately suppress long-chain acylcarnitine production in muscles, and its plasma concentration remained high during insulin clamp or a standard meal (11, 21). Thus, measurements of plasma long-chain acylcarnitine levels during glucose tolerance and insulin clamp are suitable to evaluate insulin sensitivity in muscle and the heart.

In healthy individuals, glucose-stimulated insulin release inhibits lipolysis in adipose tissue and decreases plasma FFA levels (Figure 6) (32). In our study, glucose administration induced up to a 35% decrease in FFA levels in control animals but did not induce significant decreases in plasma FFA levels in the HFD-treated or *db/db* mice. Glucose uptake measurements in both models showed substantial insulin resistance in adipose tissue. These results clearly link impaired adipose tissue insulin sensitivity with continuous FFA release in the transition from a fasted state to a fed state. A study in human subjects also demonstrated a blunted insulin-mediated decrease in plasma FFA levels after insulin clamp in patients with type 2 diabetes compared to healthy, normoglycemic, or obese individuals (11), thus supporting the clinical significance of the observed effects in this study. Overall, the decrease in plasma FFA concentrations in response to glucose administration reflects early changes in adipose tissue insulin sensitivity.

As the majority of the total acylcarnitine pool consists of acetylcarnitine (C2), the measurement of total acylcarnitine concentrations represents mainly the changes in acetylcarnitine concentrations. Moreover, the short-chain acylcarnitines are metabolites of lipids, amino acids and carbohydrates are released in plasma mainly by the liver (33), while the long- and medium-chain acylcarnitines accumulate in the tissues when the synthesis rate of acylcarnitines is not coupled with the following metabolism. We observed that short- and medium-chain acylcarnitine levels after glucose load were reduced equally in control and HFD-induced insulin resistant animals, and no consistent association between changes in plasma levels and the degree of insulin resistance was observed. Thus, short- and medium-chain acylcarnitines are not considered as markers for determining muscle or heart-specific insulin resistance.

In summary, the an insufficient decrease in plasma FFA concentrations from the fasted state to the postprandial state after glucose administration reflects even early changes in adipose tissue insulin sensitivity, while an insufficient decrease in long-chain acylcarnitine levels is associated with muscle and heart insulin resistance. The data of this study indicate that short-chain and medium-chain acylcarnitines are not promising diagnostic markers, but the measurements of changes in plasma FFA, and long-chain acylcarnitine concentrations at 2 h after glucose load in fasted subjects are useful as diagnostic markers for adipose and muscle tissue-specific insulin resistance. We propose that implementation of FFAs and long-chain acylcarnitine measurements would provide multiple clinical benefits. This diagnostic approach would provide novel possibilities to characterize tissue-specific insulin resistance during diabetes progression and intervention (both lifestyle changes and pharmacotherapy). Additionally, targeted use of treatments specific to muscle or adipose tissue could be developed based on measurements of FFAs and long-chain acylcarnitines as biomarkers. The measurements of intermediates of fatty acid metabolism might be more sensitive than fasting glucose is in the early detection of insulin resistance.

## Data Availability Statement

The datasets generated for this study are available on request to the corresponding author.

## Ethics Statement

The animal study was reviewed and approved by Latvian Animal Protection Ethical Committee, Food and Veterinary Service, Riga, Latvia.

## Author Contributions

EM, MM-K, MD, and EL: study design. MM-K, KVi, KVo, OZ-M, and EM: conducting studies. ES and SG: analytical chemistry. EM, MM-K, MD, and EL: data analysis, interpretation and manuscript preparation. All authors read and approved the final manuscript.

### Conflict of Interest

The authors declare that the research was conducted in the absence of any commercial or financial relationships that could be construed as a potential conflict of interest.

## Supplementary Material

Table S1 Acylcarnitine concentrations in fasted mice and 1h after glucose administration.

## Abbreviations

[^3^H]-DOG: [^3^H]-deoxy-D-glucose
HbA1C: glycated hemoglobin A1C
HFD: high fat diet
FAs: fatty acids
FFA: free fatty acid
GTT: glucose tolerance test.
